# High Frequency and Diversity of Tetracycline Resistance Genes in the Microbiota of Broiler Chickens in Tunisia

**DOI:** 10.3390/ani11020377

**Published:** 2021-02-02

**Authors:** Antonietta Di Francesco, Daniela Salvatore, Sonia Sakhria, Elena Catelli, Caterina Lupini, Mohamed Salah Abbassi, Ghaith Bessoussa, Salma Ben Yahia, Noureddine Ben Chehida

**Affiliations:** 1 Department of Veterinary Medical Sciences, University of Bologna, 40064 Ozzano dell’Emilia, Italy; daniela.salvatore2@unibo.it (D.S.); elena.catelli@unibo.it (E.C.); caterina.lupini@unibo.it (C.L.); 2 Institute of Veterinary Research of Tunisia, University of Tunis El Manar, Tunis 1006, Tunisia; sakhrias@yahoo.fr (S.S.); salahtoumi_mohamed@yahoo.com (M.S.A.); salmabenyahia09@gmail.com (S.B.Y.); nbenchehida@yahoo.fr (N.B.C.); 3 Commissariat Régional au Développement Agricole, Ben Arous 2063, Tunisia; ghaithbessoussa@hotmail.fr

**Keywords:** chickens, microbiota, tetracycline resistance genes, PCR, Tunisia

## Abstract

**Simple Summary:**

The extensive use of tetracyclines in clinical practice and livestock has subjected bacterial populations to selection pressure and increased the prevalence of tetracycline resistance, one of the most abundant antibiotic resistances among pathogenic and commensal microorganisms. In the present survey, DNA extracted from cloacal swabs from 195 broiler chickens in Tunisia were molecularly tested for 14 tetracycline resistance genes. A high frequency and diversity of tetracycline resistance genes in the chickens sampled were detected. The results confirm the antimicrobial resistance urgency in Tunisia’s poultry sector and suggest that the investigation of antibiotic resistance genes directly in biological samples could be a useful means for epidemiological studies on the spread of the antimicrobial resistance.

**Abstract:**

Tetracycline resistance is still considered one of the most abundant antibiotic resistances among pathogenic and commensal microorganisms. The aim of this study was to evaluate the prevalence of tetracycline resistance (*tet*) genes in broiler chickens in Tunisia, and this was done by PCR. Individual cloacal swabs from 195 broiler chickens were collected at two slaughterhouses in the governorate of Ben Arous (Grand Tunis, Tunisia). Chickens were from 7 farms and belonged to 13 lots consisting of 15 animals randomly selected. DNA was extracted and tested for 14 *tet* genes. All the lots examined were positive for at least 9 *tet* genes, with an average number of 11 *tet* genes per lot. Of the 195 animals tested, 194 (99%) were positive for one or more *tet* genes. *Tet*(L), *tet*(M) and *tet*(O) genes were found in 98% of the samples, followed by *tet*(A) in 90.2%, *tet*(K) in 88.7% and *tet*(Q) in 80%. These results confirm the antimicrobial resistance impact in the Tunisian poultry sector and suggest the urgent need to establish a robust national antimicrobial resistance monitoring plan. Furthermore, the molecular detection of antibiotic resistance genes directly in biological samples seems to be a useful means for epidemiological investigations of the spread of resistance determinants.

## 1. Introduction

Public health implications of antimicrobial resistance (AMR) are significant since the decreased effectiveness of antibiotics in treating common infections leads to an increase in the cost of health care in terms of days of hospitalization and intensive care [1]. Multiple jurisdictions, especially in Europe, have adopted mandatory restrictions on antimicrobial use. The use of antibiotics as growth promoters in animal nutrition was banned in the EU by 1 January 2006 [2] and earlier in Scandinavian countries. Otherwise, in many countries outside of Europe, the antimicrobial use in human and veterinary medicine is still unrestricted.

AMR is a current public health problem in Tunisia. In the last 15 years, the country experienced a strong increase of antibiotic resistant bacteria, from human as well as from animal or foods of animal origin, in strict relation to overuse or incorrect use of antimicrobials [3]. The Tunisian National Institute of Consumption reported that the use of antibiotics in Tunisia increased by 38% during the period between 2005 and 2013 [4]. A recent study on the trend of antibiotic use from 2000 and 2015 in 76 countries [5] placed Tunisia in second place among the most consuming countries in 2015.

With respect to the AMR concern, Tunisia has planned a national action plan for the five-year period 2019–2023, which is aligned with the WHO global action approach One Health, which integrates human health, animal health and environment [6]. In the veterinary field, the main problems consist in the scarcity of veterinary data on the procurement of antimicrobials and monitoring of their use, the fact that antibiotic therapy is frequently conducted without the bacteriological examination and without determination of the antibiotic resistance profile of the pathogen, the cost of analyses, the lack of veterinary laboratories, the usual self-medication on farms, and the purchase of antimicrobials through parallel markets.

The AMR impact in the Tunisian poultry sector is very strong, with higher resistance rates than those observed in Tunisia’s bovine and ovine industries [7]. AMR occurring in the poultry sector can spread to humans via food or water chains, environmental contamination by poultry waste or direct contacts with animals or biological substances. Both the transmission of zoonotic antibiotic resistant bacteria and of mobile genetic elements carrying genes encoding antibiotic resistance represent a public health concern, considering that antibiotics used in poultry farming may be the same, or belong to the same class, as those used in human medicine [8].

For the most part, studies on AMR in the poultry sector in Tunisia have evaluated tetracycline resistance occurrence [7,9,10,11,12,13,14,15] considering that tetracycline antibiotics are among the most commonly administered antibiotics in the commercial poultry sector worldwide [16]. Tetracycline resistance is generally caused by the acquisition of tetracycline resistance (*tet*) genes, often associated with either a mobile plasmid or a transposon. To date, at least 59 *tet* genes and 11 mosaic *tet* genes have been described [17,18]. Three main resistance mechanisms are mediated by *tet* genes: pumping the drug out of the cell before it reaches its site of action (active efflux pumps), protection of the ribosomal binding site which decreases drug binding, and enzymatic inactivation of the active compound. The first two mechanisms currently predominate in clinical settings [19].

Conventional antimicrobial susceptibility testing methods are based on bacteriological culture and antibiotic susceptibility testing of the isolated microorganisms. Recent studies introduced an exclusively molecular approach, investigating the presence of antibiotic resistance genes directly examined in biological [20,21,22] or environmental [23] samples. A limitation of culture independent methods is the inability to determine which bacterial species the resistance genes originate from. On the other hand, these methods, in addition to the advantage of speed, avoid a possible underestimation of AMR occurrence due to a consistent nonculturable fraction of microorganisms. Singer et al. [24] suggested that, due the capability of bacteria to transfer resistance genes, analysis of AMR emergence, dissemination and persistence might be better conducted at the gene level. Considering AMR genes as contamination markers, methods which allow searching for these genes rather than for the bacteria carrying them could help in epidemiology to analyze the spread of resistance determinants [25,26].

The aim of this study was to evaluate the presence of 14 tetracycline resistance genes in DNA samples from cloacal swabs of 195 broiler chickens sampled at two slaughterhouses in Tunisia.

## 2. Materials and Methods

### 2.1. Sampling

From February to March 2019, individual cloacal swabs from 195 broiler chickens were collected at two slaughterhouses in the governorate of Ben Arous (Grand Tunis, Tunisia). Chickens belonged to 13 lots from 7 farms (A–G), located in 5 governorates (Ben Arous, Bizerte, Béja, Zaghouan and Nabeul), in a perimeter of 60 km. Each lot consisted of 15 animals randomly selected. All the farms were industrial, except for one rural chicken farm (Farm E/Lot 7).

### 2.2. Molecular Analysis

#### 2.2.1. DNA Extraction

Total DNA was extracted from each cloacal swab using the QIAamp DNA mini kit (Qiagen, Hilden, Germany) following the supplier’s recommendations. One extraction control was also included, consisting of kit reagents only.

#### 2.2.2. DNA Amplification and Sequencing

DNA samples were investigated by PCRs to search 14 genes involved in the three tetracycline resistance mechanisms: the tetracycline efflux pumps [*tet*(A), *tet*(B), *tet*(C), *tet*(D), *tet*(E), *tet*(G), *tet*(K), *tet*(L), *tetA*(P)], the ribosomal protection [*tet*(M), *tet*(O), *tet*(Q), *tet*(S)], and the enzymatic inactivation [*tet*(X)].

Each *tet* gene was amplified by an individual PCR, using primers described by Ng et al. [27] (Table 1).

Different PCR protocols were carried out: 5 min of initial denaturation at 94 °C followed by 35 cycles at 94 °C for 1 min; 55 °C [*tet*(A), *tet*(C), *tet*(G), *tet*(L) and *tet*(O)], 50 °C [*tet*(K)], 51 °C [*tet*(P) and *tet*(S)], or 53 °C [*tet*(B), *tet*(D), *tet*(E), *tet*(M), *tet*(Q) and *tet*(X)] for 1 min; and 72 °C for 1 min. A final step of 10 min at 72 °C completed the reaction. The DNA extracted from *Escherichia coli* field strains, containing tetracycline resistance plasmids, was used as a positive control. The extraction control and a distilled water negative control were also included.

The PCR products were analyzed by gel electrophoresis (1% agarose); the DNA bands were stained with ethidium bromide and were then visualized using ultraviolet (UV) trans illumination. The amplicons were purified using the High Pure PCR Product Purification Kit (Roche, Mannheim, Germany), and both DNA strands were sequenced (Bio-Fab Research, Rome, Italy). The sequences obtained were compared with the public sequences available using the BLAST server in GenBank (National Center for Biotechnology Information 2019).

## 3. Results

All the lots examined were positive for at least nine *tet* genes, with an average number of 11 *tet* genes per lot. The *tet*(A), *tet*(B), *tet*(K), *tet*(L), *tet*(M), *tet*(O), *tet*(Q), *tet*(S), and *tet*(X) genes were found in 100% of the lots. Of the 195 animals tested, 194 (99%) were positive for one or more *tet* genes (Table 2).

**Figure 1:**
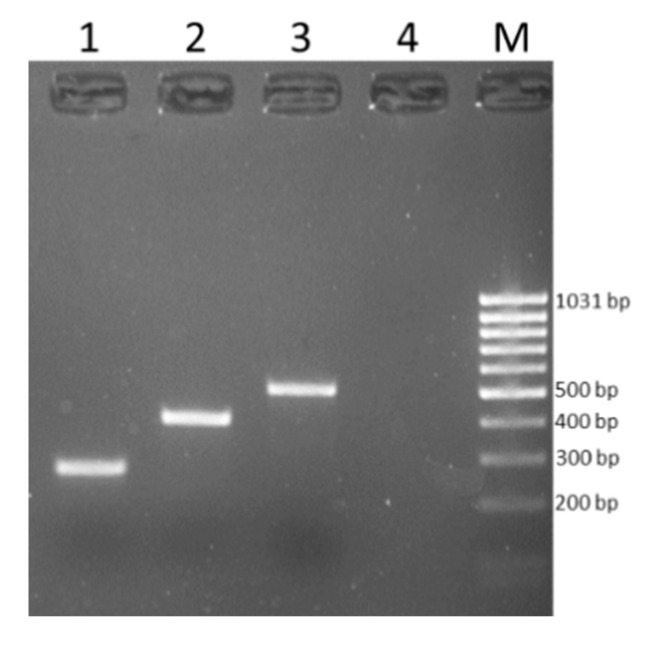
PCR amplicons. Lane 1, 267 bp *tet*(L) gene fragment; lane 2, 406 bp *tet*(M) gene fragment; lane 3, 515 bp *tet*(O) gene fragment; lane M, MassRuler Low Range DNA Ladder, (Thermo Fisher Scientific, Vilnius, Lithuania).

With respect to the *tet* gene frequencies, *tet*(L), *tet*(M), and *tet*(O) genes were found (each) in approximately 98% of the samples (Figure 1), followed by *tet*(A), *tet*(K), and *tet*(Q) genes, which were found in 90.2%, 88.7%, and 80% of samples, respectively. *Tet*(C) (27.7%), *tet*(D) (18.4%), *tet*(P) (7.7%), *tet*(E) (2.5%), and *tet*(G) (0.5%) genes were detected at low frequencies.

For each *tet* gene amplified, with the exception of the *tet*(E) gene, the identity of the amplicons was confirmed by the comparison between the sequence obtained and the corresponding sequences from antibiotic resistant Gram-positive or Gram-negative bacteria in the GenBank database, showing 99–100% nucleotide similarity. Sequencing failed for *tet*(E) amplicons, probably because the 5 positive samples showed a low amplification signal. One sequence for each of the 13 *tet* genes successfully sequenced was deposited in the Gen-Bank database under accession numbers MW079481–MW079493.

## 4. Discussion

The results of the present study showed high rates of tetracycline resistance genes in the chicken lots examined and were 100% positive for at least 9 of the 14 *tet* genes tested.

Interestingly, a high gene diversity for antibiotic resistance was highlighted, with *tet*(L), *tet*(M), and *tet*(O) genes exhibiting the highest rates of occurrence among the *tet* genes tested in all sampled lots. With respect to the *tet*(L) gene, Roberts [19] reported, in the last years, a very large increase in the number of genera carrying *tet*(L) gene, up to the current 47 among Gram-positive and Gram-negative genera [28,29]. The prevalence of *tet*(M) gene was consistent with other reports showing a wide distribution of this gene, probably because of its association with conjugative chromosomal elements [19]. Conjugative transposons appear to have less host specificity than do plasmids, which may explain the detection of *tet*(M) in 80 different genera including 39 Gram-positive and 41 Gram-negative genera [28,29]. The *tet*(O) gene has been detected in 19 Gram-positive and 20 Gram-negative genera [28,29]. This gene has been found on plasmids [30] as well as in association with functional conjugative transposons [31]. Interestingly, our results showed a high (72.3%) frequency of *tet*(X) gene, which is responsible for the enzymatic inactivation of the tetracycline molecule. Until now, *tet*(X) has been found in only Gram-negative genera, except Cutibacterium genus [28,29]. Little research has been conducted on *tet*(X) because this gene was not considered clinically relevant. However, recent studies suggested that *tet*(X) could be useful in the screening of various environmental contexts [32,33]. Finally, the result obtained for *tet*(G), detected in only one sample, was not surprising given the low prevalence reported in literature [34].

The *tet* gene frequencies observed in the backyard chickens (Lot 7/Farm E) was comparable to that highlighted in the industrial poultry of the other lots, although the overuse of antibiotics is more common in industrial production. On the other hand, AMR is a complex topic attributable to many factors other than the medical administration of antimicrobials. These include the following: (i) most antimicrobial agents are produced by strains of fungi and bacteria that occur naturally in all environments, including soil [35]; (ii) bacteria may also acquire resistance determinants through horizontally mobile elements including conjugative plasmids, integrons, and transposons [36]; and (iii) the agricultural use of antimicrobial agents selects for antibiotic resistance, as antibiotics persist in soil and aquatic environment [37].

To our knowledge, this is the first time a high number of *tet* genes was investigated in food animals in Tunisia. Previous surveys have mainly focused on *tet*(A), *tet*(B), or *tet*(C) genes in *E. coli* isolates of animal origins, with *tet*(A) and *tet*(B) results predominant [9,10,11,12,13]. However, in some of these studies, *tet* genes were not detected in tetracycline resistant bacterial isolates [10,12], probably due to the few *tet* genes investigated. Otherwise, Klibi et al. [14] testing *tet*(K), *tet*(L), *tet*(M), *tet*(O), and *tet*(S) genes in enterococcal isolates from poultry and beef/sheep meat highlighted a higher gene frequency for *tet*(M) and an almost total correspondence between antibiotic susceptibility testing and *tet* gene molecular detection.

Our molecular investigation confirmed a high rate of tetracycline resistance in the Tunisian poultry sector according to previous culture-based studies performed in Tunisia [7,9,10,11,12,13,14,15]. A high frequency of *tet* genes not commonly investigated was highlighted, suggesting the usefulness of a more extensive molecular approach than that usually applied in order to avoid false negative results.

## 5. Conclusions

Our findings confirm AMR as a serious threat for human health and food production in Tunisia. National surveillance of the antibiotic resistance of animal origin, awareness of the good practices of antibiotic therapy in veterinarians, breeders, and pet owners, and strict control of antibiotic trade appear essential to ensure a successful action plan.

Regarding the molecular approach used in this study, the results suggest that the investigation of antibiotic resistance genes directly in biological samples could be a useful tool, for epidemiological purposes, to analyze the spread of resistance determinants.

## Figures and Tables

**Table 1:** Tetracycline-Resistant PCR Primers [27].

Tetracycline Resistance Gene	PCR Primer Sequence 5′–3′	Amplicon Size (bp)
*tet*(A)	GCT ACA TCC TGC TTG CCT TC’ CAT AGA TCG CCG TGA AGA GG	210
*tet*(B)	TTG GTT AGG GGC AAG TTT TG GTA ATG GGC CAA TAA CAC CG	659
*tet*(C)	CTT GAG AGC CTT CAA CCC AG ATG GTC GTC ATC TAC CTG CC	418
*tet*(D)	AAA CCA TTA CGG CAT TCT GC GAC CGG ATA CAC CAT CCA TC	787
*tet*(E)	AAA CCA CAT CCT CCA TAC GC AAA TAG GCC ACA ACC GTC AG	278
*tet*(G)	GCT CGG TGG TAT CTC TGC TC AGC AAC AGA ATC GGG AAC AC	468
*tet*(K)	TCG ATA GGA ACA GCA GTA CAG CAG ATC CTA CTC CTT	169
*tet*(L)	TCG TTA GCG TGC TGT CAT TC GTA TCC CAC CAA TGT AGC CG	267
*tet*(M)	GTG GAC AAA GGT ACA ACG AG CGG TAA AGT TCG TCA CAC AC	406
*tet*(O)	AAC TTA GGC ATT CTG GCT CAC TCC CAC TGT TCC ATA TCG TCA	515
*tet*(S)	CAT AGA CAA GCC GTT GAC C ATG TTT TTG GAA CGC CAG AG	667
*tet*(P)	CTT GGA TTG CGG AAG AAG AG ATA TGC CCA TTT AAC CAC GC	676
*tet*(Q)	TTA TAC TTC CTC CGG CAT CG ATC GGT TCG AGA ATG TCC AC	904
*tet*(X)	CAA TAA TTG GTG GTG GAC CC TTC TTA CCT TGG ACA TCC CG	468

**Table 2:** Number of cloacal swabs PCR positive for the 14 tested tetracycline resistance (*tet*) genes encoding tetracyclines resistance (Each lot consisted of 15 animals).

Lot/Farm	Active Efflux									Ribosomal Protection				Enzymatic Inactivation
	*tet*(A)	*tet*(B)	*tet*(C)	*tet*(D)	*tet*(E)	*tet*(G)	*tet*(K)	*tet*(L)	*tet*(P)	*tet*(M)	*tet*(O)	*tet*(Q)	*tet*(S)	*tet*(X)
1/A	14	13	1	1	0	0	13	13	0	13	14	13	9	13
2/A	15	14	4	2	1	0	14	15	0	15	15	15	12	12
3/B	15	13	2	6	1	0	15	15	0	15	15	15	8	14
4/C	14	11	8	0	0	0	15	15	0	15	15	14	11	15
5/D	14	11	3	0	0	0	13	15	1	15	15	15	2	8
6/C	15	10	8	6	3	1	13	15	1	15	15	12	10	12
7/E	11	12	6	7	0	0	15	15	8	15	15	14	2	6
8/D	11	11	8	7	0	0	15	15	4	15	15	14	6	9
9/F	13	10	0	0	0	0	12	15	0	14	15	11	7	11
10/D	15	11	1	0	0	0	12	13	1	15	15	6	1	9
11/F	13	10	6	6	0	0	11	15	0	15	15	12	2	12
12/D	12	8	7	1	0	0	12	15	0	15	14	9	4	10
13/B	14	15	0	0	0	0	13	15	0	15	14	6	9	10
Total N (%)	176 (90.2)	149 (76.4)	54 (27.7)	36 (18.4)	5 (2.5)	1 (0.5)	173 (88.7)	191 (98)	15 (7.7)	192 (98.4)	192 (98.4)	156 (80)	83 (42.5)	141 (72.3)

## Data Availability

The sequences generated in this study are available in Genbank under accession numbers MW079481-MW079493.
